# Growth in children on kidney replacement therapy: a review of data from patient registries

**DOI:** 10.1007/s00467-021-05099-4

**Published:** 2021-06-18

**Authors:** Marjolein Bonthuis, Jérôme Harambat, Kitty J. Jager, Enrico Vidal

**Affiliations:** 1 ESPN/ERA-EDTA Registry, Department of Medical Informatics, Amsterdam Public Health Research Institute, Amsterdam UMC, University of Amsterdam, Meibergdreef 9, J1B-108.1, P.O. Box 22700, 1100 DE Amsterdam, The Netherlands; 2 Department of Pediatrics, Bordeaux University Hospital, Bordeaux Population Health Research Center UMR 1219, University of Bordeaux, Bordeaux, France; 3 Division of Pediatrics, Department of Medicine, University of Udine, Udine, Italy

**Keywords:** Children, Growth, Final height, Chronic kidney disease, Kidney replacement therapy, Dialysis, Transplant

## Abstract

Growth retardation is a major complication in children with chronic kidney disease (CKD) and on kidney replacement therapy (KRT). Conversely, better growth in childhood CKD is associated with an improvement in several hard morbidity–mortality endpoints. Data from pediatric international registries has demonstrated that improvements in the overall conservative management of CKD, the search for optimal dialysis, and advances in immunosuppression and kidney transplant techniques have led to a significant improvement of final height over time. Infancy still remains a critical period for adequate linear growth, and the loss of stature during the first years of life influences final height. Preliminary new original data from the European Society for Paediatric Nephrology/European Renal Association-European Dialysis and Transplant Association (ESPN/ERA-EDTA) Registry confirm an association between the final height and the height attained at 2 years in children on KRT.

## Introduction

Chronic kidney disease (CKD) has wide-ranging and long-term consequences for children and their families, including growth retardation, which is a marker of disease severity. Growth retardation may occur at any stage of CKD and is related to multiple modifiable and non-modifiable risk factors [1]. The latter include age at onset of CKD, abnormal birth history, kidney disease etiology, genetic factors (i.e., parental height), and use of required medications (i.e., steroids for immune-mediated diseases or after kidney transplantation) [2]. Primary pathogenic mechanisms can involve prenatal/perinatal factors, nutrition and caloric intake, mineral and bone metabolism disorders, hormonal influences from the pituitary gland, and other metabolic abnormalities (collectively termed as *uremic milieu*), including metabolic acidosis, anemia, and inflammation.

Clinical and experimental evidence demonstrates that perturbations in the growth hormone (GH)-insulin-like growth factor-1 (IGF-1) axis are responsible for many important complications seen in CKD, such as growth retardation and protein energy wasting, as well as disease progression [1, 3, 4]. These alterations include changes in the 24-h levels of spontaneous GH release, reduced GH receptor density in target organs, and disturbed cascade of intracellular signaling events (post-receptor defect). This results in a functional IGF-1 deficiency, in which free bioactive levels are further reduced by an increased binding capacity due to excess of IGF-binding proteins. Growth hormone insensitivity in CKD lays the basis for recombinant human growth hormone therapy (rhGH), an effective and well-tolerated intervention to improve growth.

Adequate physical growth is a paramount outcome in children with CKD. Better growth in childhood CKD is associated with an improvement in several hard clinical endpoints [5–9]. Moreover, adequate stature during pediatric CKD influences patient and parental perspectives [10, 11], and satisfaction with adult life [12].

Given the overall impact of growth on health-related quality of life (HRQoL), evaluation, prevention, and management of growth failure are essential in children with CKD. Trends in physical growth are commonly evaluated in registry-based research and might indicate a change in the epidemiology of childhood CKD.

In this review, we aim to give an overview of the statural growth in children receiving chronic kidney replacement therapy (KRT), integrating data from international registries. Moreover, prompted by a suggestion from Professor Lesley Rees during a recent scientific meeting, we used data from the European Registry for Children on KRT (European Society for Paediatric Nephrology/European Renal Association-European Dialysis and Transplant Association (ESPN/ERA-EDTA) Registry) to evaluate the hypothesis that height attained during infancy in stage 5 chronic kidney disease (CKD 5) patients is a predictor of final height.

## Association of growth retardation with outcomes

Growth failure after pediatric KRT has been associated with poor social and health outcomes. In pre-dialysis populations, HRQoL was lower among children with short stature [11, 13], and height gains and rhGH use were associated with improved parent-reported HRQoL [11]. In a recent analysis from the Kids with CKD study, among 375 children from Australia and New Zealand with CKD, on dialysis and after kidney transplantation (KT), quality of life scores were significantly lower among dialysis patients, and short stature was associated with poorer quality of life [14]. Children with short stature more often have emotional and behavioral problems, including anxiety, low self-esteem, social immaturity, learning disabilities, and poor academic achievement [15, 16]. One-third of adult patients with childhood onset CKD 5 were dissatisfied with their body height [17], and a Dutch cohort study found that patients starting KRT < 15 years of age who attained a short adult height were less likely to have children after 30 years on KRT [18]. Furthermore, short stature has been associated with lower levels of employment and lower marital status [12]. However, a more recent study on adult social and professional outcomes after pediatric KT did not find such associations, and the authors concluded that height seemed to be sufficiently improved and is no longer a major determinant in social and professional outcomes after pediatric CKD 5 [19].

Short stature could also affect kidney graft function. In the CKiD cohort, patients with a short stature prior to transplantation had a 40% shorter time to reaching an eGFR < 45 ml/min/1.73 m^2^ after KT compared to children with a normal stature, although this association was partly explained by socio-economic status, disease severity, and mid-parental height [20].

Finally, short stature in pediatric CKD 5 has also been associated with increased hospitalization [7] and mortality [7, 9, 21], mainly due to a higher risk of infectious complications. Although not causally associated, the elevated mortality risk in CKD 5 patients with short stature may in part reflect poor nutritional status and severity of illness. Still, the associations of growth failure with poor patient outcomes indicate the importance of obtaining a normal adult height for the patients’ daily functioning. Table 1 summarizes the associations of height in children on KRT and various outcome measures reported by several international pediatric KRT registries.

**Table 1:** Associations between height and different outcome measures: results from international registries

Outcome measure	Registry database	Result
Morbidity (hospitalization rate)	USRDS [5]	Increased risk of hospitalization in case of growth failure: 1.8 hospitalizations/patient year (py) in severe growth failure, 1.74 hospitalizations/py in moderate growth failure, and 1.2 hospitalizations/py for normal growth subjects over 5 years of follow-up. After adjustment, patients with severe (HR: 1.12, 95% CI: 1.03–1.22) and moderate (HR: 1.26, 95% CI: 1.17–1.36) growth failure had higher hospitalization rates than those with normal growth
	NAPRTCS [7]	Patients with short stature (height SDS < −2.5 SDS) had significantly more hospital days per month than patients with height ≥ −2.5 SDS (median 0.73 compared with 0.44, *p* < 0.001)
Mortality	USRDS [9]	Risk of death was higher in children with short (< 3^rd^ percentile) (HR: 1.49, 95% CI: 1.33–1.66) and tall (> 97^th^ percentile) (HR: 1.32, 95% CI: 1.03–1.69) stature at KRT initiation.
	NAPRTCS [7]	Children initiating dialysis with height SDS < −2.5 were more likely to die than patients with height in the normal range (HR: 2.07, 95% CI: 1.53–2.79)
Final height	NAPRTCS [22]	Incidence of retarded final height SDS (<−1.88) was increased in patients with low height SDS at transplantation (OR: 0.39, *p* = 0.001)
	ESPN/ERA-EDTA [6]	Height SDS at start of KRT was positively associated with final height SDS. Adjusted final height SDS was 0.37 (95% CI: 0.32–0.41) higher per 1 SDS increase in height SDS at KRT
	ESPN/ERA-EDTA (Unpublished data, 2021)	Height SDS at 2 years seemed positively associated with final height SDS (*ß* = 0.17, *p* = 0.05) (Fig. 2)

## Improvement in growth after KRT

By the 1970s, the availability of KRT allowed many children with CKD 5 to survive into adulthood. Other clinical outcomes than patient survival then became critical, and special focus was put on growth in children with CKD in the following decades. Franke et al. have reported the gains made in growth and maturation in pediatric CKD 5 patients, particularly in Germany [23]. In the mid-1980s, the mean height standard deviation score (SDS) of 732 European children on KRT included in the EDTA registry was < −3. Mean height SDS markedly improved over time to −1.80 in 384 German children receiving KRT in the 1998–2009 era [23]. The final height also improved significantly over the past decades [6, 24]. In the USA, final height SDS for transplanted patients having reached 19 years (*n* = 2569) improved from −1.93 in 1987–1991 to −0.89 in the 2007–2013 period [24]. Similarly, final height SDS increased from −2.06 SDS in European children on KRT who reached adulthood in 1990–1995 to −1.33 SDS in those reaching adulthood in 2006–2011 [6]. However, the ESPN/ERA-EDTA Registry showed no secular trends in growth post transplantation in European children between 1990 and 2012 according to the period of KT, suggesting that the improvement of final height over time is most likely explained by better pre-CKD 5 care [25].

## Dialysis

In pediatric patients, dialysis should be considered as a transition period before receiving a KT and should aim at attaining adequate body weights for receiving a KT. In CKD patients, growth failure is an indication for dialysis initiation, and good growth and development during dialysis are the principal indices of dialysis adequacy. “Adequate dialysis” usually refers to a minimum dose below which a clinically unacceptable rate of negative outcomes occurs, and it is defined by a fixed threshold or a number. Given the strong association between growth, clinical outcomes, and quality of life in children receiving KRT, “adequate growth” becomes a marker of “optimal dialysis” in this setting.

In the 2011 annual dialysis report from the NAPRTCS registry [26], the 5022 patients had on average a height SDS of −1.60 at dialysis initiation. Height deficits were worse for males, younger patients, and those treated with hemodialysis (HD) as compared with peritoneal dialysis (PD). Patients were also stratified according to a baseline height deficit: height SDS < or > than the third percentile of the normal population. Children with a height SDS > 3^rd^ percentile at baseline experienced a decrease in height SDS during the dialysis course, whereas the height SDS of children with a height SDS < 3^rd^ percentile at baseline improved slightly from −3.21 SDS to −2.90 SDS by 24 months. In all age categories, children treated with HD experienced a worse median change from baseline during the dialysis course, as compared with PD.

### Residual kidney function

Residual kidney function (RKF) exerts a significant influence on growth in children on dialysis and is a better predictor of longitudinal growth than dialytic clearance. In 24 patients treated with PD for a minimum of 1 year [27], the mean height SDS changed from −1.58 at baseline to −1.78 after 1 year of treatment. Catch-up growth was observed in 9 patients (37%), 7 of whom had RKF. In contrast, only 5 of 15 patients (33%) with a negative delta height SDS had RKF. Similarly, an analysis of 12-month follow-up data in 214 pre- and early-pubertal patients in the International Pediatric Peritoneal Dialysis Network (IPPN) database showed that preserved residual diuresis was significantly associated with the odds of increasing height SDS in children treated with PD (odds ratio: 3.25; 95% confidence interval: 1.66–6.31) [28].

### Peritoneal dialysis

There are few papers describing growth outcomes in infants on chronic PD, and the results from these studies are controversial. In the cohort studied by Ledermann et al. [29], mean height SDS at the start of PD was −1.8, and it increased to −1.1 at 1 year and to −0.8 at 2 years. The majority of the 20 children in this case series were affected by CAKUT and had preserved RKF. None of the infants received rhGH therapy, while 18 were given enteral feeding via a nasogastric tube or percutaneous gastrostomy. In a sample of 84 infants who started PD at < 1 year of age, the Italian Registry of Chronic Dialysis showed that mean height SDS was −1.65 at the start of chronic PD, −1.82 after 12 months, and −1.53 after 24 months [30]. The longer follow-up in this study allowed demonstration of a catch-up growth in half of the treated infants, and SDS values at 2 years of follow-up were similar to those at PD initiation. Infants with catch-up growth were not different from those without, with regard to several biochemical parameters, RKF, distribution of comorbidities, and proportion of children on rhGH treatment. Interestingly, a positive relationship was found between height SDS and both fill volume and treatment duration after 1 year of PD. Moreover, children with better growth rates suffered from a significantly lower number of peritonitis episodes.

Among 153 children from the IPPN Registry who initiated PD during infancy, the use of biocompatible PD fluids low in toxic glucose degradation products was consistently associated with better body growth [31]. The association prevailed even after correction for nutritional status and region, and was quantitatively stronger than the effect of gastrostomy feeding.

These findings suggest that the management of growth failure in children on PD can be optimized using a maximum dialytic and nutritional approach and by examining all suitable strategies for preserving diuresis, reducing peritoneal membrane inflammation, and preventing peritonitis. Dialysis prescription should not be based on the worsening of biochemical parameters but instead should reflect a timely tailoring to body size, RKF, and metabolic needs.

### Hemodialysis

In children on chronic HD, despite reaching an adequate dialysis dose based on urea kinetic modeling, many children do not show improvement of appetite, weight gain, or statural growth. With conventional HD, significant dietary and fluid restrictions are needed to prevent interdialytic weight gain during the “off dialysis” days. However, implementing these restrictions becomes challenging, especially in the pediatric age group. Clinical signs of underdialysis indicate that the prescription should be optimized. HD intensification relies on modification of factors such as duration, frequency, time, and modality (HD versus hemodiafiltration (HDF)). In a pilot study by Fischbach et al. [32], five oligoanuric dialysis patients were converted from standard HDF (4 h, three times/week) to daily online HDF (3 h, six times/week). After 6 months, the only prepubertal child included showed a significant catch-up growth, resulting in a height SDS gain of 1.5 over 24 months. De Camargo et al. performed an observational study on 24 children with CKD 5 undergoing daily HD [33]. The authors found that the intensified prescription favored a 0.5 SDS height gain in one-third of patients without rhGH treatment as compared with 8% in a control group of 26 children on a concurrent conventional HD regimen. In a large multi-center observational study comparing outcomes of conventional HD versus post-dilution online HDF in children [34], a significant increase in height SDS over 1 year of treatment was found in patients on convective therapy, whereas height SDS remained stable in conventional HD. Convective therapies might achieve higher clearance of middle molecules, including IGF-1 binding proteins that contribute to functional IGF deficiency and relative rhGH insensitivity.

## Kidney transplantation

Growth after KT is mainly affected by the degree of pre-transplantation height deficit, age at KT, graft function, and steroid exposure.

### Pre-KRT growth

Growth determinants during the pre-KRT period may include age at diagnosis of CKD, nutritional intake, primary kidney disease, tubular impairment, CKD–mineral bone disorder (CKD–MBD), hormonal disorders, and pre-transplantation use of rhGH and steroids. Two periods are at a particular risk of impaired growth velocity: infancy and puberty. Height velocity generally decreases once GFR is below 25 ml/min/1.73 m^2^ [35]. However, there is evidence that growth impairment already starts earlier. Indeed, in a CKD analysis of the NAPRTCS [36] including 1901 patients with CKD stage 2 (GFR of 50–75 ml/min/1.73 m^2^), 22% had height SDS below the third percentile (≤ −1.88), and in 2477 children with CKD stage 3 (GFR of 25–50 ml/min/1.73 m^2^), 37% had a height SDS ≤ −1.88. Pre-KT growth impairment may be reduced by adequate conservative treatment of CKD, including aggressive nutritional support during infancy and early childhood [37] and treatment with rhGH when indicated [4]. This has resulted in a significant improvement of pre-KT growth. Data from the NAPRTCS has shown a remarkable improvement in height SDS at the time of first KT, which increased from a mean of −2.4 in 1987 to −1.4 SDS in the 2008 cohort [38].

### Age at transplantation

**Fig. 1:**
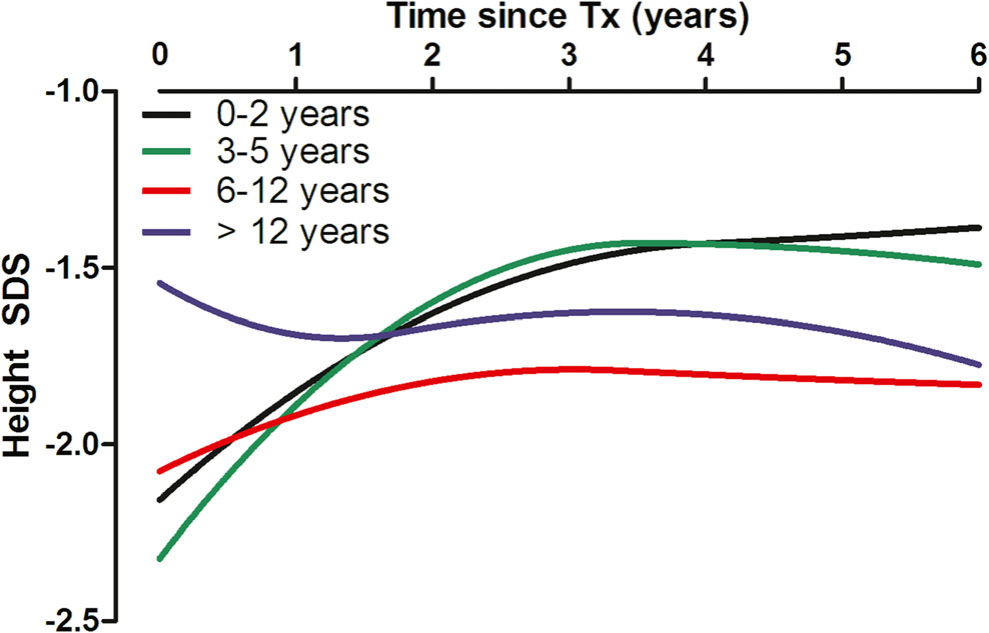
Unadjusted post-transplant growth patterns stratified by age at kidney transplantation. (Reproduced with minor revision from [25], used with permission)

In an ESPN/ERA-EDTA report [25], only infants and pre-school age children (2 to 5 years) exhibited catch-up growth post-transplantation (Fig. 1). However catch-up growth occurred mainly during the first 3 years post-transplantation, and no further improvement was observed thereafter; pre-school age children who had the largest deficit at the time of KT (< −2 SDS) exhibited the best growth improvement of 0.6 to 0.8 SDS. Conversely, school age children (6 to 12 years) and adolescents demonstrated either a limited improvement in height SDS or even no catch-up growth after KT (Fig. 1). Similar growth patterns have been reported by the NAPRTCS [38]. The prepubertal growth deceleration that occurs in the normal population is prolonged after KT, and puberty and bone age are usually delayed [39, 40]. This means that growth continues for longer than normal, but the height gain is rarely as much as expected due to loss of height potential [39, 41]. However, some authors have reported that normal pubertal development and significant catch-up growth can occur post-KT even in children of pubertal age [42, 43].

### Kidney graft function

The effect of a reduced GFR on growth has been known for a long time [44]. A report from the NAPRTCS assessed final adult height in recipients transplanted before 11 (girls) or 12 (boys) years, showing that a decreased GFR was an independent predictor of reduced final height [22]. Guest et al. reported that prepubertal children with a serum creatinine above 120 μmol/L did not exhibit catch-up growth in the first year post-KT [45]. Nissel et al. showed that prepubertal catch-up growth and total pubertal height gain correlated positively with GFR [39]. Data from the NAPRTCS found that graft function at 1 and 12 months was associated with growth after KT; those with a GFR > 90 ml/min/1.73 m^2^ showed improved height SDS over time [38]. An analysis by eGFR group 12 months after transplantation showed similar results. More recent studies confirmed that graft function is a major determinant of growth after KT [25, 46].

### Steroid therapy

Since its introduction more than 60 years ago, it has become clear that daily and prolonged steroid therapy leads to growth impairment [47]. Steroid therapy inhibits growth through interferences with the hypothalamus–pituitary/growth hormone/insulin-like growth factor axis and by its direct effect on bone formation. It has also been suggested recently that steroids may decrease longitudinal bone growth in pediatric KT recipients by stimulating the FGF23/FGFR3 signaling pathway [48]. Pharmacokinetic studies of methylprednisolone in pediatric liver and kidney transplant recipients have demonstrated that area under serum concentration–time (AUC) rather than dose was predictive for growth retardation [49, 50]. However, this correlation between AUC and growth was not unanimously found in transplanted children treated with prednisone or prednisolone [51]. Observational and registry data showed that steroid exposure is associated with post-transplant height SDS [25, 52]. There have been studies in the 1990s suggesting an improvement in growth with daily low or alternate-day steroid therapy [53, 54]. Steroid withdrawal/avoidance protocols in pediatric KT have been associated with a significant improvement in height SDS in randomized controlled trials [55–59]. Prepubertal recipients appeared to have the greatest benefit which seems to be only very limited in pubertal children (Table 2).

**Table 2:** Summary of RCTs of steroid withdrawal/avoidance and the effects on growth

Author	Year	Follow-up (months)	Intervention	Steroid withdrawal/avoidance	Change in height SDS			Controls	Change in height SDS
				N	Overall	Prepubertal	Pubertal		
Hocker [55]	2010	24	Late withdrawal	23	0.60	0.70 (*n* = 13)	0.40 (*n* = 7)	19	−0.20
Benfield [56]	2010	30	Late withdrawal	73	0.16			59	−0.04
Sarwal [57]	2012	36	Avoidance	60	−0.92	−0.43 (*n* = 11 < 5 years)		70	−0.96
Mericq [58]	2013	12	Early withdrawal	14	1.20	1.30 (*n* = 12)		16	0.60
Webb [59]	2015	24	Early withdrawal	98	0.57	0.69 (*n* = 48)	−0.04 (*n* = 50)	98	0.33

### Donor type

Pape et al. identified 51 boys who received a KT (30 deceased donors and 21 living related donors) before the rhGH era and were followed for at least 5 years [60]. Children who received a living donor kidney had a significantly greater height SDS and growth velocity in the first 5 years post-transplantation than those who received a graft from a deceased donor. Interestingly, this difference remained significant after adjustment for potential confounders, including graft function. In the NAPRTCS, living donor kidney recipients had a reduced height deficit compared to that of deceased donor kidney recipients, and this difference persisted after transplantation, but the mean adjusted difference was less than 0.3 SDS at 5 years post-KT [38]. In the ESPN/ERA-EDTA Registry, children who received a deceased donor kidney were shorter by 0.15 SDS at KT and tended to show a lower catch-up growth post-transplant than those transplanted with a living donor (+ 0.35 SDS deceased vs. + 0.39 SDS living) [25].

### Preemptive kidney transplantation

As dialysis is associated with decreased growth velocity, preemptive KT might optimize growth outcome. Some studies suggested better height SDS in the first years post-transplantation in those children who received a preemptive KT compared to those with dialysis prior to KT [38, 43, 61]. However, this finding was not confirmed by others after adjustment for potential confounders [46, 52].

### Other factors

A previous NAPRTCS report suggests that race/ethnicity may also have an impact on growth following transplantation [62]. The change in height SDS was negative in African–American and Hispanic recipients but positive in Caucasians. However, ethnicity did not significantly influence growth in multivariable analysis.

Birth parameters are important determinants of post-transplant growth. Children born small for gestational age (SGA) have significantly poorer growth outcome after KT compared with non-SGA patients, and their growth is more likely to be affected by other factors such as use of rhGH in the pre-transplant period, preemptive KT, and graft function [63].

Finally, several clinical factors like primary kidney disease (congenital CKD, syndromic disease), anemia, high blood pressure, metabolic acidosis, or vitamin D deficiency have variable associations with height SDS and growth post-KT [25, 46, 52, 64].

## Final height

Despite adequate metabolic and nutritional control, intensified dialysis regimens, and successful KT, reduced final height (below the third percentile for age and sex) is present in approximately 40% of pediatric CKD 5 patients [6, 23]. The median final height ranges from 145 to 162 cm in females and from 156 to 174 cm in males [6, 24, 39, 65–67]. In Europe, the median (IQR) final height after childhood KRT was −1.65 (−2.64; −0.78) SDS and did not significantly differ between boys and girls [6], whereas the NAPRTCS registry reported a median final height of −1.23 SDS [24], a difference most likely caused by the use of different reference charts [68].

Important determinants of final height are older age at start of KRT and KT, cumulative time with a functioning graft, and greater height SDS at KRT initiation and at KT [6, 39, 65, 69] (Table 1). Additionally, post-transplant catch-up growth is restricted to the youngest patients (< 6 years at KT) [25, 69], and there was no improvement in post-KT height SDS in the past 25 years in Europe, suggesting that better growth management in the pre-CKD 5 period is mainly responsible for the improvement in final height. The pre-existing height deficit thus seems to be one of the most important factors for the final height of pediatric CKD 5 patients.

**Fig. 2:**
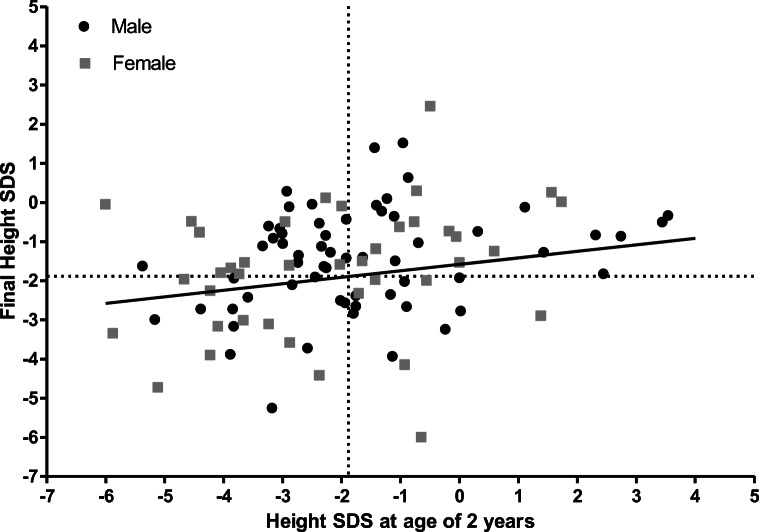
Final height standard deviation scores (SDS) by height SDS at the age of 2 years among 101 patients on kidney replacement therapy (KRT) from the ESPN/ERA-EDTA Registry. The solid line depicts the linear regression analysis adjusted for age and period of KRT, sex, and cause of kidney failure

In line with this, we hypothesized that the height of young children with CKD 5 is already predictive for their final height. To study the association between height SDS at the age of 2 years and final height SDS, we used data from the ESPN/ERA-EDTA Registry. Data was available for 101 patients from 13 European countries (60% male, 41% congenital anomalies of the kidney and urinary tract, median age at KRT (IQR): 1.7; 0.8–2.5 years). Height SDS was expressed according to national or European growth charts [68]. Linear regression analysis was used, and we adjusted for potential confounders. Indeed, after adjusting for age and period at KRT initiation, sex, and cause of kidney failure, there was a trend toward an association between height SDS at the age of 2 years and final height SDS (*ß* = 0.17, *p* = 0.05) (Fig. 2) (Unpublished data ESPN/ERA-EDTA Registry, 2021). However, further research on a larger number of subjects (including those not (yet) on KRT) should reveal whether height at young age is truly predictive of final height in pediatric CKD 5.

The current final height of CKD 5 patients remains suboptimal, but strategies such as steroid avoidance/withdrawal immunosuppressive regimens in KT recipients [65, 66] and more regular use of rhGH [70, 71] may improve final height, possibly even when started during puberty [67]. The expected increase in final height after being treated with rhGH for a period of 2–5 years approximates 7 cm [4]. However, unfortunately rhGH prescription seems to be limited and mostly determined by physician and patient attitudes toward rhGH therapy rather than by financial hurdles [72].

## Role of nutrition

Inadequate nutrition is a common cause of growth failure in children with CKD. Reduced appetite, gastroesophageal reflux, and vomiting are important factors contributing to poor nutritional intake [73]. Poor appetite has several potential causes and may result from reduced taste sensation, which worsens when CKD progresses [74], restrictive diets, medication use, protein losing states, polydipsia [75], and chronic inflammation [76].

Growth in early childhood is crucial for final height potential as one-third of postnatal statural growth occurs during the first 2 years of life. In infants and young children, poor nutrition is the most important factor contributing to growth impairment [77].

Recently, the Pediatric Renal Nutrition Taskforce published clinical practice guidelines for energy and protein requirements for children on CKD [78]. In CKD 2–5D, energy intake should approximate that of healthy children of similar chronological age, and in case of suboptimal growth or impaired weight gain, energy intake should be adjusted toward the higher end of the suggested dietary intake. If children are not able to meet nutritional requirements, tube feeding should be initiated [78]. Indeed, infants treated with PD in the IPPN registry who received tube feeding, especially those on gastrostomy feeding, experienced better preservation of growth than those on demand feeding: when considering periods of at least 6 months, the median change in length SDS was −1.35 (2.63) SDS/year during periods of demand feeding, −0.72 (1.59) SDS/year during nasogastric tube feeding, and −0.50 (2.47) SDS/year during periods of gastrostomy feeding. Length SDS at last observation was significantly higher in patients with gastrostomy feeding as compared with those on demand and/or nasogastric tube feeding, possibly because of reduced vomiting in case of gastrostomy [31]. Whether tube feeding is associated with improvement in height SDS beyond infancy is controversial, although there are some studies showing catch-up growth during prepubertal years in CKD patients when tube fed [37]. A more recent study among 50 children with CKD and on dialysis not treated with rhGH or KT demonstrated that enteral tube feeding started after the age of 2 years in prepubertal children (2–11 years) was associated with an improved height of +0.21 SDS after 2 years of tube feeding. However, improvements were restricted to children younger than 6 years of age and those not treated with dialysis [79].

On the other hand, tube feeding is effective in improving BMI SDS [31, 79]. Paralleling the global obesity epidemic, overweight and obesity are also emerging problems in the pediatric CKD population [80–82]. According to the ESPN/ERA-EDTA Registry, 20.8% of children < 16 years on KRT were overweight, and 12.5% were obese [81]. In patients from the CKiD cohort, the reported median energy and protein intake among children with CKD was high, and at least half of the children consumed more energy and protein than recommended [83, 84].

Energy requirements and enteral feeding regimens should be adjusted in order to promote optimal growth and to prevent obesity. Surprisingly, both the IPPN Registry and the ESPN/ERA-EDTA Registry found an association between short stature and obesity. These findings may reflect previous attempts to correct growth failure by energy-dense nutrition, in fact leading to overweight and obesity with very limited effect on height gain.

Nevertheless, optimal nutrition is important during all phases of growth in pediatric CKD, and successful nutritional management requires involvement of a pediatric renal dietician [85, 86].

## rhGH treatment

Despite supportive measures aimed at correcting complications of CKD and optimization of KRT, some children with CKD or KT recipients cannot approach normal height without rhGH therapy [2]. The GH insensitivity in CKD can be overcome by the administration of supraphysiological doses of rhGH, which results in increased circulating levels of free IGF-1 and promotes longitudinal growth.

A Cochrane systematic review based on 16 randomized controlled studies (enrolling 809 children) demonstrated that 12 months of 28 IU/m^2^/week rhGH in children with all clinical settings of CKD (conservative treatment, on dialysis, or after KT) resulted in a 3.88 cm increase in height velocity above that of untreated patients [87]. The frequency of reported side effects of rhGH was generally similar to that of the control group. Efficacy of rhGH therapy was further confirmed by observational data from the NAPRTCS registry [88]. A total of 787 children with CKD previously rhGH naïve who received rhGH for 1–4 years were paired with 787 control patients. Treatment with rhGH resulted in a significantly greater height velocity SDS, with no significant impact on the BMI or kidney function.

Recently, the ESPN CKD-MBD, Dialysis and Transplantation Working Groups published detailed clinical practice recommendations for the use of rhGH in children with CKD, on dialysis and after KT [4]. According to these recommendations, all children with stage 3–5D CKD, who have a well-defined growth failure despite correction of any amenable complications of CKD, should be candidates for rhGH therapy, provided they still have a growth potential. The same indication is provided for KT recipients who have not experienced any spontaneous catch-up growth 1 year after transplantation and a steroid-free immunosuppressive regimen is not feasible.

Despite supporting evidence, there are still many obstacles to rhGH therapy in children with CKD 5. Short stature might be perceived as a cosmetic issue only, and it is easy to ignore. This “lack of urgency” may result in a delayed prescription of rhGH therapy, thus reducing treatment efficacy [89]. Treatment costs and reimbursement mechanisms might also represent a barrier to care, but an ESPN/ERA-EDTA Registry study found patient and clinician attitudes toward rhGH therapy a bigger issue than costs [72]. Earlier initiation of rhGH in a small child could be more cost-effective, as it might also improve the patient body weight, thus allowing early KT and reducing the time on long-term dialysis.

Infants with CKD from their first months of life often have difficulty maintaining adequate nutrition, which contributes to the high prevalence of short stature in this population. The IPPN Registry analyzed data from 153 children in 18 countries who commenced chronic PD at < 24 months of age [31]. In multivariate analysis, the administration of rhGH for at least 6 months was independently associated with improved length, even after adjusting for regional factors. Clinical experience supports the use of rhGH also in infants with CKD. The ESPN clinical practice guidelines recommend that rhGH therapy should be considered for infants older than 6 months of age with CKD 3–5D who have a poor growth despite provision of adequate nutrition [4].

## Key summary points

i) Despite treatment advances in pediatric nephrology care growth retardation remains a major problem in patients treated for childhood CKD 5.
ii) Most improvements are observed in the pre-CKD 5 period, but optimization of dialysis care, steroid avoidance/withdrawal regimens post-transplantation, and adequate use of rhGH treatment might be helpful to further decrease the burden of growth failure in children on KRT.
iii) According to our preliminary ESPN/ERA-EDTA Registry analysis, appropriate growth management during infancy is particularly critical, since patient final height seems associated with height attained at 2 years of age.

### Multiple choice questions (answers are given after the reference list)

1. Which statement about growth in pediatric KRT patients is correct?
  - Improvements in height are mainly obtained during the pre-CKD 5 period
  - rhGH should not be initiated during infancy
  - Enteral feeding is only advised in dialysis patients
  - Height is not associated with increased hospitalization rates
2. Which factors are associated with better growth in pediatric patients treated with dialysis?
  - Biocompatible PD fluids
  - Hemodiafiltration
  - Residual kidney function
  - All of the above
3. Which patient group showed the poorest catch-up growth after kidney transplantation?
  - Infants
  - Pre-school children (2–5 years)
  - 6–12-year-old patients
  - Adolescents
4. How many pediatric KRT patients roughly grow up as stunted adults (i.e., have a reduced final height)?
  - 30%
  - 40%
  - 50%
  - 60%
5. According to the recent ESPN clinical practice guidelines on rhGH which patients would be eligible for rhGH therapy?
  - Dialysis patients with growth failure
  - All children with CKD, on dialysis and after kidney transplantation with growth failure
  - CKD and dialysis patients with growth failure
  - All children with CKD stage 3–5D with growth failure once other potentially treatable risk factors for growth failure are adequately addressed, and there is growth potential, as well as KT patients without spontaneous catch-up growth 1-year post-KT and if steroid-free immunosuppression is not possible.
